# Addressing Clinician-Educator Hesitancy Toward Artificial Intelligence Through a Peer-Led Instructional Design

**DOI:** 10.7759/cureus.113342

**Published:** 2026-07-25

**Authors:** Nathan Wilson

**Affiliations:** 1 Pediatrics, Augusta University, Augusta, USA

**Keywords:** artificial intelligence in healthcare, artificial intelligence in medicine, educator training program, faculty development in medical education, teaching technology

## Abstract

Introduction: Artificial intelligence (AI) has had a tremendous impact on medical education. National data are indicative of massive clinical interest and usage, but a "faculty readiness gap" exists among experienced clinician-educators. Though closing this gap is essential, traditional training models often fail to improve appropriate application of AI tools. This study describes medical education development, innovation, and competency in AI (MEDIC-AI), an instructional method designed to increase faculty readiness through peer modeling of AI applications critical to success.

Methods: A physician-educator (NW) at the Medical College of Georgia (MCG) at Augusta University created a peer-led instructional platform to explain and model the use and application of AI tools. The pilot episode was distributed anonymously to colleagues, and perceived usefulness (PU), perceived ease of use (PEOU), and behavioral intention (BI) to implement at least one AI tool within 30 days were measured using a cross-sectional survey based on the Technology Acceptance Model (TAM).

Results: Evaluations from 33 faculty demonstrated substantial acceptance, reporting high PU (90.9%) and PEOU (81.8%) regarding the format, and 87.9% demonstrated BI. Qualitative data suggest that peer explanation and modeling were critical to the results.

Conclusion: Using AI can simplify workflow for busy physician-educators. When AI applications are simply and concisely explained and demonstrated by a trusted colleague hosting a do-it-yourself (DIY) format, participants reported increased usefulness, ease of use, and intent to implement. This suggests that incorporation of peer leadership into traditional instruction on AI tool use has the potential to close the "faculty readiness gap," improving teaching and patient care.

## Introduction

Artificial intelligence (AI) has had the comparable impact of a tsunami on medical education, leaving seasoned clinicians with a stark choice - ride the wave or be crushed by it [1]. For generations, experienced and committed clinician-educators have inspired learners with foundational medical knowledge, often while sharing clinical wisdom, stories, and practical tips for helping patients. Faculty who are now in mid-career or later can remember paper charts and life before the internet. These clinicians have witnessed the explosion of AI, yet many experience a "faculty readiness gap" in terms of competency and integration of these tools into teaching and clinical care [2]. While young faculty and trainees are natively technology literate, older attending physicians often report limited confidence in understanding, evaluating, or utilizing emerging digital tools [2,3].

This readiness gap is reflected in the American Medical Association’s (AMA) 2026 Physician Survey on Augmented Intelligence, where 92% of respondents expressed a desire for more AI training, yet only 11% described their existing training as "adequate" [4]. Standard solutions offered by teaching institutions frequently fail to bridge this gap [2,5]. These modalities at times produce cognitive overload and trigger "expert self-consciousness" among senior clinicians who feel intimidated by the speed of technological change [3,6]. Some clinical faculty with much to offer may fall into self-protective silence, lacking the confidence to continue the vital flow of generational wisdom to future physicians.

In searching for ways to increase faculty readiness, the author (NW) created medical education development, innovation, and competency in AI (MEDIC-AI) [7]. Its novelty and strength lie in its peer-led design - a trusted colleague demonstrating ease of use. The purpose of this report is to evaluate institutional acceptance and behavioral impact of MEDIC-AI among clinician-educators at the Medical College of Georgia at Augusta University (MCG), utilizing the Technology Acceptance Model (TAM) framework to measure perceived usefulness, ease of use, and behavioral intention in an attempt to understand whether an authentic, peer-led approach can influence user confidence and willingness to integrate AI tools into daily teaching and clinical responsibilities [8].

This study has been accepted for presentation as an abstract at the 2026 AAMC Learn Serve Lead Annual Meeting, to be held on November 6, 2026.

## Materials and methods

MEDIC-AI design

The author designed MEDIC-AI to produce tailored faculty development that fits into a busy physician educator's schedule [1]. Core domains of MEDIC-AI were mapped to the Accreditation Council for Graduate Medical Education (ACGME) systems-based practice milestones on clinical technology and efficiency workflows, and to the AAMC Foundational Competencies on digital health literacy and data management, with a focus on practice efficiency and technological adaptation [9,10]. The present design is influenced by national consensus that data science literacy can no longer be treated as elective but must be embedded directly as a core competency in medical training [11].

This project used an insider-researcher methodology. The author, as a mid-career clinician-educator at MCG, possessed personal understanding of colleagues' educational and clinical stressors, permitting highly nuanced and contextualized guidance regarding opportunities for AI tool application and integration (e.g., drafting letters of recommendation {LoRs}, preparation of PowerPoint presentations for pre-clerkship student lectures, and clinical documentation), tasks that can be so technologically burdensome that they can tempt senior faculty to lose their joy in teaching [2,12].

Pilot episode production

To create the pilot episode, references were compiled from national consensus statements, peer-reviewed medical education literature, reports on LLM usage and application, and global AI ethical frameworks using OpenEvidence (Miami, FL: OpenEvidence, Inc.) [12-15]. Production was streamlined and intentionally simplified, using only Google's NotebookLM (Mountain View, CA: Google LLC) and an Apple iPhone 14 (Cupertino, CA: Apple Inc.).

The references were uploaded to NotebookLM, and a default-length Deep Dive audio overview (approximately 14 minutes) was generated. The author, who also served as host, recorded institutionally relevant commentary using the Voice Memo iPhone application, sandwiching the AI-generated podcast between a human peer intro and outro using the iMovie application [1]. To reflect a do-it-yourself (DIY) level of competency rather than a studio-caliber production, the AI-generated content was used without editing, sound engineering, or phonetic corrections [1]. Minor quirks (e.g., the AI podcast hosts mispronouncing "ACE inhibitors" and "LORs") were preserved [1]. The final product was a human-AI hybrid - a 17-min pilot episode that discussed potential uses of AI in medical education.

Pilot and post-survey distribution

In early 2026, an invitation to participate was extended internally to 96 clinician-educators at MCG using email listservs from the author's department, pre-clerkship course instructors, curriculum office leadership, and faculty development leadership. To minimize bias that could occur because of the author's relationship with potential respondents, the pilot episode and post-survey were accessed via an anonymous link. No personal identifiers, IP addresses, or metadata were collected, and informed consent detailing anonymity, voluntary participation, and no penalty for nonparticipation was required before completing the survey. With consideration of limited participants invited, a quantitative/qualitative survey design was chosen and distributed only after institutional review board (IRB) approval was obtained [16,17]. Of 96 invited faculty, 33 listened to the pilot and completed the survey, yielding a response rate of 34.4%. The survey was mapped to domains of the TAM, evaluating the following three variables on a five-point Likert scale: perceived usefulness (PU), perceived ease of use (PEOU), and behavioral intention (BI) to implement at least one AI tool within 30 days (Table 1) [8]. Quantitative metrics were evaluated via percentage-agreement frequency distributions, and qualitative responses were analyzed using thematic coding to capture respondents' underlying psychological barriers and motivation [18].

**Table 1:** MEDIC-AI survey questions. PU: perceived usefulness; PEOU: perceived ease of use; BI: behavioral intention; LoRs: letters of recommendation; MEDIC-AI: medical education development, innovation, and competency in AI

Item	Type	Domain	Survey question/prompt
Q1	Quantitative (five-point Likert)	PU	This 'pro-hybrid' (Dr. Wilson + AI) podcast exposed me to novel uses of AI for administrative tasks (e.g., LoRs) or clinical teaching.
Q2	Qualitative (open text)	PU	Describe one clinical or admin task (e.g., drafting letters of recommendation, patient education, or documentation) where you see the most immediate potential for AI to reduce your cognitive load.
Q3	Quantitative (five-point Likert)	PEOU	The podcast format made complex AI concepts easy to digest compared to traditional faculty development workshops.
Q4	Qualitative (open text)	PEOU	In terms of the 'learning curve' for AI tools, what technical or conceptual barrier feels most daunting to you as a medical educator? How did the 'human-in-the-loop' podcast format (Dr. Wilson hosting) affect your confidence in navigating that barrier?
Q5	Quantitative (five-point Likert)	BI	I intend to use at least one AI tool discussed in this episode within the next 30 days.
Q6	Qualitative (open text)	BI	As you consider integrating AI into your workflow for teaching, what is your primary ethical or professional concern regarding its impact on medical student or resident learning?

## Results

Quantitative evaluation (TAM domains)

**Figure 1:**
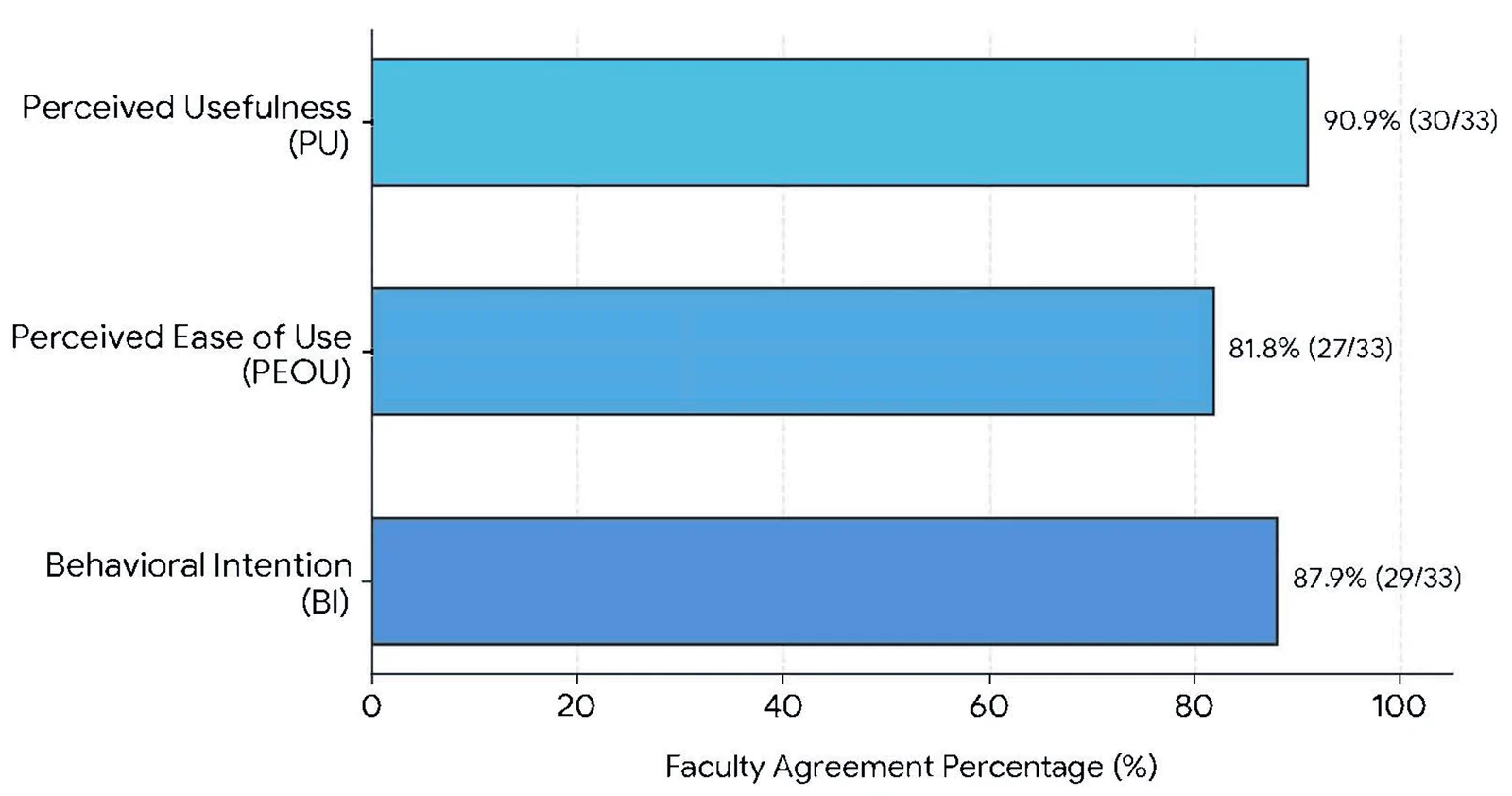
TAM metrics for MEDIC-AI pilot (n=33). MEDIC-AI: medical education development, innovation, and competency in AI; TAM: Technology Acceptance Model

Anonymous data collected from 33 respondents showed high acceptance across all primary TAM variables as follows: PU - 90.9% of respondents (n=30/33) agreed that MEDIC-AI exposed them to novel uses of AI for administrative tasks and clinical teaching. PEOU - 81.8% of respondents (n=27/33) reported that the pilot episode made complex AI concepts easier to digest compared to traditional faculty development workshops. BI - 87.9% of respondents (n=29/33) reported intent to use at least one discussed AI tool within 30 days (Figure 1).

Qualitative thematic analysis

The six-phase approach established by Braun and Clarke was followed for qualitative analysis [18]. After familiarization with responses, initial codes were generated. Codes for actions and processes dominated in question two (e.g., documentation, help, preparation, save time), while codes for emotions were better suited to questions four and six (e.g., overwhelmed, afraid, concerned). Initial codes were categorized, and themes were generated (e.g., help with admin tasks, human helps trust, worried about student AI over-reliance). Initial themes were broadened and refined to better reflect the entire dataset and the respondents' emotions. Finally, the following themes emerged (Table 2).

**Table 2:** MEDIC-AI survey qualitative responses. MEDIC-AI: medical education development, innovation, and competency in AI; MSPEs: medical student performance evaluations; LoRs: letters of recommendation; UTD: UpToDate; CV: curriculum vitae

Theme	Description	Representative faculty responses (n=33)
Theme 1: the demand for administrative relief	Student success (LoRs, MSPEs, CV summary), preparation of teaching materials, practice efficiency, and documentation	"It would be great to have help summarizing CVs of students asking for LoRs." "I spend a lot of time educating patients and could see using AI to design something that would communicate my ideas without me taking the time each visit." "I wonder if AI can help us schedule better. We have time slots of different lengths; there's a lot of inefficiency that is worsened with cancellations." "Documentation would be much less burdensome with AI." "I would like to be able to make PowerPoint slides faster for teaching familiar topics."
Theme 2: peer hosting is important for AI trust and engagement	Improving technological reluctance, openness to further peer-led engagement, and desire for guided instruction on prompt engineering	"Just trying the new stuff. As someone my age and stage, Dr. Wilson encouraged me that it's possible to master these tools." "Well, if Nathan can do it, maybe I can also." "Whether to trust the AI-generated content has been daunting as a provider; Dr. Wilson generated more confidence." "The speed that new tools are coming out is intimidating. He is reassuring, and his use of a tool to build the podcast is pretty cool." "It was nice to have the human in the loop. I would be interested in seeing exactly how it was done. What are the steps and prompts used to create something like a podcast?"
Theme 3: pedagogical and ethical concerns	Concerns for learners (intellectual shortcuts, erosion of critical thinking, losing ability to empathize) environmental impact	"I want to understand more how we can guard against potential learner abuse of these tools." "Professional concern remains how to keep educators in charge of education and updating/ensuring the quality of AI teaching tools." "Making sure students learn that humanistic approach to patient care." "Originality. Are students using AI as an additional tool in their belt like a textbook, lecture, or UTD? Or are they using AI as a replacement for their own critical thinking?" "I want the residents and students to not lose their ability to talk and empathize by over-relying on technology." "I am ethically concerned about the environmental impact of using AI. The energy need for data centers is immense, and we seem to be using AI as a 'toy' without any concern for the environmental impact."

Demand for Administrative Relief

Many faculty expressed feeling overwhelmed and focused heavily on using AI to reduce clerical time. They listed multiple tasks common to the clinician-educator experience, recognizing the possibility for AI to help and an openness to learning. Medical student performance evaluations (MSPEs), summarizing student CVs for letters of recommendation (LoRs), and aiding clinical tasks (e.g., documentation, scheduling, and patient education) were all mentioned [16].

Peer Hosting is Important for AI Trust and Engagement

Multiple responses suggested that the author's role as host improved baseline technological reluctance. Others expressed that hearing a colleague gave them more confidence to try new AI applications even as they recognized a need for help understanding how to optimize output from the AI [16].

Pedagogical and Ethical Concerns

Despite an eagerness to use AI for personal administrative relief, faculty expressed deep concern regarding learner usage, mostly centered on trainees utilizing AI as an intellectual shortcut, echoing national worry that these tools may bypass foundational clinical reasoning and diagnostic critical thinking [19]. Other responses focused on data hallucination and the potential depersonalization of humanistic patient care, concerns other authors have voiced [3,6,20]. Additionally, one respondent expressed ethical concern about the environmental impact of AI use.

## Discussion

This study suggests that shifting faculty development from impersonal AI training to a peer-led model might improve competency and integration of important new AI technologies among older clinician-educators. While traditional, commercial training modalities can produce reluctance and fatigue, this pilot suggested that imperfect tools and operators might be beneficial even in the absence of a professionally produced podcast when a trusted colleague is involved. Understanding of context and shared experience gives the peer host the ability to empathize with and tailor to the institutional audience. For example, in the MEDIC-AI pilot episode, the author related a personal lesson from a community-impacting event to encourage listener use of AI tools [1]. Institutional identification of seasoned educators and their subsequent strategic involvement in required training modules would enable similar commentary, perhaps increasing trust, usage, and integration of otherwise daunting modalities.

In addition to the data represented in this study, the author was contacted informally by several older colleagues stating they would benefit from visual guidance detailing the methods for using the AI tools described in the pilot. In response, MEDIC-AI was transformed from audio-only into a DIY video series (MEDIC-AI on YouTube) [21]. Coworker feedback also urged consideration of the needs of non-educator, community-based physicians, suggesting creation of episodes demonstrating customizable educational podcasts, user-sourced slide deck generation, billing and coding, tailored patient education handouts, among others.

Limitations

The author acknowledges limitations of the study. First, this project was conducted as a single-institution study within a specific geographic region at MCG, which may limit the generalizability to other academic environments. Second, the modest sample size (n=33) reflects an early-adopter cohort recruited through internal listservs, potentially introducing selection bias toward faculty members more inclined toward digital innovation. A single coder was used for qualitative analysis, limiting reliability. Additionally, the study contained no control group or pre-intervention data for comparison. Finally, this study relied on a cross-sectional assessment of 30-day behavioral intentions rather than long-term tracking of AI tool mastery or downstream measurement of learner educational outcomes.

## Conclusions

As clinician-educators age and technology advances ever more rapidly, there is a danger of losing the transfer of wisdom and knowledge that has benefited generations of physicians. This study suggests that traditional faculty development might be improved through peer-led models to bridge the "faculty readiness gap" for AI use in medical education, though results should be interpreted with caution given the single-institution design and small sample size. Broader application lies in the reproducible DIY format, demonstrating an adaptable framework for institutions working to improve AI literacy across graduate and undergraduate medical training. Altering learner experience through a trusted peer-led environment may assist modern clinician-educators in embracing and integrating emerging AI technology. Future piloting should include multiple educational institutions, longitudinal implementation, and competency metrics to further understand whether peer-led models are more beneficial.

## References

[1] Wilson N. MEDIC-AI Pilot Episode 1 (2026). MEDIC-AI episode 1 final. Medical College of Georgia Institutional Internal Broadcast. Audio Resource Published April.

[2] (2025). Medical schools train faculty on AI best practices. https://www.aamc.org/news/medical-schools-train-faculty-ai-best-practices.

[3] Saroha S (2025). Artificial intelligence in medical education: promise, pitfalls, and practical pathways. Adv Med Educ Pract.

[4] American Medical Association Center for Digital Health and AI (2026). 2026 physician survey on augmented intelligence. AMA.

[5] Pillay R (2026). The AI revolution in medical education. J Educ Pract Trends.

[6] Maaß L, Grab-Kroll C, Koerner J (2025). Artificial intelligence and ChatGPT in medical education: a cross-sectional questionnaire on students' competence. J CME.

[7] Wilson N MEDIC-AI: a scalable faculty development model for integrating artificial intelligence competencies across undergraduate and graduate medical education. https://drive.google.com/file/d/1MjnrzfIrMwp2P_xh81TLKIsYWzvy03fa/view?usp=sharing.

[8] Davis FD (1989). Perceived usefulness, perceived ease of use, and user acceptance of information technology. MIS Quart.

[9] Edgar L, Hatlak K, Haynes IL, Holmboe ES, Hogan SO, McLean S (2026). The Milestones Guidebook: Competency-Based Medical Education and Milestones Development. https://www.acgme.org/globalassets/milestonesguidebook.pdf.

[10] (2026). Artificial intelligence competencies across the learning continuum. DC: AAMC Competency-Based Medical Education Hub.

[11] Seth P, Hueppchen N, Miller SD, Rudzicz F, Ding J, Parakh K, Record JD (2023). Data science as a core competency in undergraduate medical education in the age of artificial intelligence in health care. JMIR Med Educ.

[12] (2024). Augmented intelligence development, deployment, and use in health care. Digital Medicine Briefing.

[13] Suresh S, Misra SM (2024). Large language models in pediatric education: current uses and future potential. Pediatrics.

[14] Oregon Health & Science University (OHSU (2026). School of medicine policy. Graduate Medical Education Policy Manual: Policy.

[15] (2021). Ethics and Governance of Artificial Intelligence for Health: WHO Guidance. https://hash.theacademy.co.ug/wp-content/uploads/2022/05/WHO-guidance-Ethics-and-Governance-of-AI-for-Health.pdf.

[16] Wilson N MEDIC-AI pilot data set. https://drive.google.com/file/d/13IHgqtZOY6vJy7bLuqamQhwHKsiYEUKj/view.

[17] Augusta University Institutional Review Board (2026). A scalable faculty development model for integrating artificial intelligence competencies across undergraduate and graduate medical education. https://drive.google.com/file/d/1J_z3AEpGvbfzCH6r6m_FS99UkLm-49vk/view.

[18] Braun V, Clarke V (2006). Using thematic analysis in psychology. Qual Res Psychol.

[19] Nguyen T (2024). ChatGPT in medical education: a precursor for automation bias?. JMIR Med Educ.

[20] Ba H, Zhang L, Yi Z (2024). Enhancing clinical skills in pediatric trainees: a comparative study of ChatGPT-assisted and traditional teaching methods. BMC Med Educ.

[21] Wilson N (2026). MEDIC-AI. https://www.youtube.com/@MEDIC-AI.

